# Personalised prevention: what patients and citizen advocates want for better engagement – a qualitative study

**DOI:** 10.1186/s12889-025-24925-0

**Published:** 2025-11-04

**Authors:** Loes Lindiwe Kreeftenberg, Lidewij Henneman, Claudia Louati, Yasemin Zeisl, Estefanía Callejas de Luca, Daniela Quaggia, Martina C. Cornel, Carla G. van El

**Affiliations:** 1 Department of Human Genetics, Amsterdam UMC, location Vrije Universiteit Amsterdam, Amsterdam, the Netherlands; 2 Amsterdam Public Health Research Institute, Amsterdam, the Netherlands; 3 European Patients’ Forum (EPF), Brussels, Belgium; 4 Cittadinanzattiva APS - Active Citizenship Network (ACN), Rome, Italy

**Keywords:** Patient and public engagement, Community participation, Stakeholder engagement, Empowerment, Personalised prevention, Precision health, Personalised medicine, Public health, Best practices, Strategy recommendations

## Abstract

**Background:**

Incorporating public and patient perspectives is essential to advancing personalised prevention. Personalised prevention focuses on preventing disease onset, progression, and recurrence by tailoring interventions on the basis of an individual’s biological, environmental, behavioural, socioeconomic, and cultural characteristics. This study explored what patients and the public want for better engagement and empowerment in prevention, aiming to develop key considerations across three domains (Research, Care, and Governance), and offering practical points to consider for improving personalised prevention strategies.

**Methods:**

In cocreation with the European Patients Forum (EPF) and Cittadinanzattiva APS-Active Citizenship Network (ACN), semi-structured individual interviews and focus groups were conducted with 29 participants, comprising of 17 citizen advocates and 12 patients (including advocates) across 16 European countries, with experience in seven distinct disease groups. The participants were recruited through ACN and EPF via newsletters and mailing lists. Thematic analysis was performed via MAXQDA software. This study adhered to the Consolidated Criteria for Reporting Qualitative Research (COREQ).

**Results:**

Findings were clustered into three key themes for better engagement in personalised prevention: (i) Information and Communication, where patients and the public emphasised the need for clear and accessible health information and user-friendly digital platforms; (ii) Representation and Inclusivity, highlighting calls for inclusive research, community engagement, and mental health integration; and (iii) Ethical and Regulatory Considerations, with concerns over equity and the potential shift from solidarity-based care to individual risk assessment, underscoring the need for robust privacy protection and equitable policies.

**Conclusions:**

Enhancing patient and public engagement in personalised prevention requires more focus on communication, inclusivity, and secure data use. The findings provide actionable insights, promoting systematic engagement across Research, Care, and Governance. Clear information about prevention strategies and treatment options must be accessible, while diverse voices should be represented in decision-making. Collaboration with communities and better use of patient data can enhance prevention efforts. Policies should ensure ethical implementation, address data protection, and promote equity, transparency, and patient and public empowerment in healthcare, ultimately fostering a more inclusive approach to personalised prevention.

**Supplementary Information:**

The online version contains supplementary material available at 10.1186/s12889-025-24925-0.

## Background

Personalised prevention is an emerging approach within public health that aims to prevent the onset, progression and recurrence of disease through the adoption of targeted interventions that consider biological information (e.g., genetics, demographics, health conditions), environmental and behavioural characteristics, and the socioeconomic and cultural context of individuals [1]. The vision of personalised prevention as part of a broader personalised medicine programme relies on empowering and engaging patients and the public [2]. The idea is that when people understand their health and recognise potential risks, individuals can take informed action to prevent disease from occurring or their health deteriorating [1–4]. For example, awareness of genetic predispositions or lifestyle factors allows individuals to adopt preventive measures tailored to their specific risks. This concept aligns with the definition of empowerment from the European Union Joint Action on Patient Safety and Quality of Care (PaSQ), which describes empowerment as a multifaceted process that helps individuals gain control over their own lives and expand their ability to take meaningful action on matters that they deem significant [5]. Patient empowerment is therefore considered pivotal in enhancing care outcomes in personalised prevention and healthcare. According to Steele et al., patient empowerment is seen as a process of “activating” patients, moving away from the passive ‘sick role’, and instead choosing to take control and responsibility for their healthcare decisions [6].

For the broader public, patients and their families to be active partners in healthcare, they must be systematically and meaningfully engaged in the planning, delivery and evaluation of the following domains relevant for personalised prevention: Research, Care and Governance. In the Research domain, the term public engagement has been used to characterise patient and public contributions to research via roles that range from “passive” study participants to “active” members involved in all phases of the research [7], including agenda setting, prioritisation, selection of outcome measures, and dissemination of results. In the Care domain, public and patient engagement aims to increase individuals’ active participation in their healthcare and disease prevention [8, 9]. This may include, the development of patient information. Finally, in the Governance domain, the involvement of the public and patients in decision-making processes regarding personalised prevention policies and programmes encompasses their participation in policy development, guideline formulation, and organisational governance structures [10], for instance, decision-making on disease-specific centres of expertise. By actively engaging the public and patients, governance processes become more transparent, accountable, and responsive to the needs and perspectives of the communities they serve [11].

This study aims to explore what patients and the public want for better engagement and empowerment and how to meaningfully and effectively engage and empower patients and the wider public in personalised prevention. Semi structured individual interviews and focus groups were conducted to gather insights across three domains (Research, Care, and Governance) and identify practical points to consider for improving personalised prevention strategies. Engagement methods, such as co-design workshops, focus groups, and advisory panels, vary in suitability depending on contextual factors and objectives impacting the level of personalised prevention, for instance whether the aim is to consult participants or co-create e.g. a care practice [12]. Varying forms and levels of engagement can enhance the impact of patients and broader public engagement in personalised prevention [13]. Therefore, our findings indicate no single best practice for empowerment, but support context-specific approaches which may be further dependent on the scale of engagement, e.g. at a regional, national or even international level.

The incorporation of citizen and patient engagement insights is essential for raising awareness of personalised prevention’s potential to improve - individual and population level - health outcomes. The development of key considerations across Research, Care, and Governance can provide practical recommendations for enhancing these personalised prevention strategies. Collecting first-hand feedback from individuals about their personalised prevention and engagement experiences is vital for refining implementation. Inclusive stakeholder engagement fosters trust among physicians, researchers, patients, and the wider public, ensuring that personalised prevention initiatives are responsive to community needs [8].

This study contributes to the European PROPHET project which aims to cocreate a ‘PeRsOnalised Prevention roadmap for the future HEalThcare’ with stakeholders to support the implementation of innovative, sustainable and high-quality personalised approaches that are effective in preventing chronic diseases [1].

## Methods

### Ethics approval

The Medical Ethics Review Committee of Amsterdam University Medical Center reviewed the study protocol and decided that the Medical Research Involving Medical Subjects Act (WMO) does not apply to this study and conforms to relevant Dutch research regulations (no. 2024.014).

### Study design and interview guide

**Fig. 1:**
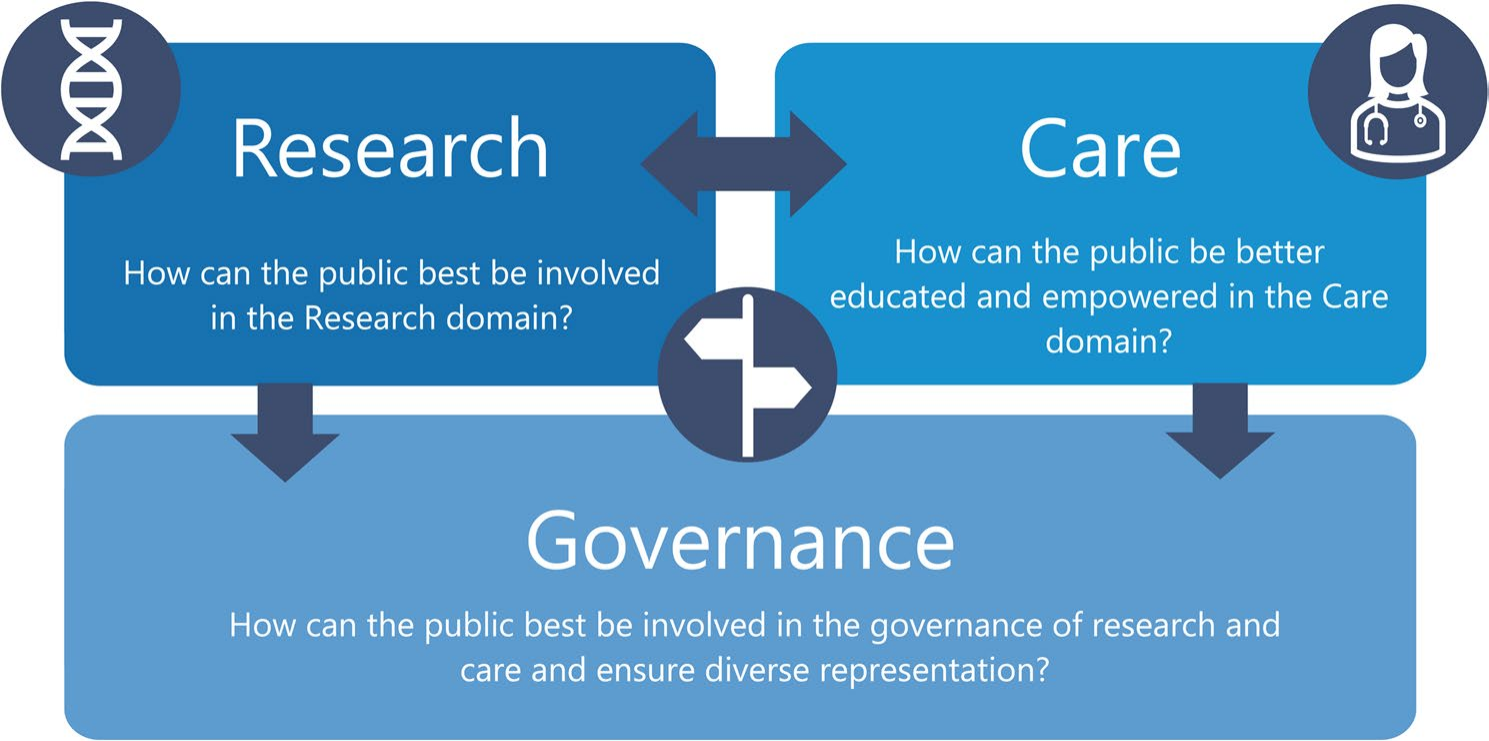
Domains of Research, Care and Governance in Personalised Prevention

A qualitative research strategy was chosen not only to obtain the opinions of the citizen advocates and patients on active and meaningful engagement in personalised prevention but also to explore how personal experiences shape their opinions. We ensured a mix of online focus groups (group-based approach) as well as individual interviews to explore patient and citizens perspectives on improving engagement and empowerment in personalised prevention. A semi-structured interview guide (see Supplementary Material 1), was developed to gather insights on enhancing engagement, communication, education, representation, and empowerment, structured around three key domains relevant to personalised prevention: Research, Care, and Governance (Fig. 1). The three domains were selected based on converging trends in public and patient engagement in healthcare and its governance, and the rise of personalised medicine as a model to increase the use of data and research in person-centred care [13–15]. The questions in this study were developed on the basis of prior work mapping engagement strategies through a scoping review of relevant literature, which helped shape the key themes and focus areas for the discussions [13]. To report the findings, we adhered to the COREQ checklist [16].

### Participants

A purposive sampling strategy was used to recruit the participants, conducted by the European Patients’ Forum (EPF) and Cittadinanzattiva - Active Citizenship Network (ACN) via newsletters and emails to their mailing lists and networks. At EPF, participants were assigned to either a focus group or an interview on the basis of their role as patients or patient advocates, ensuring that discussions were tailored to the specific perspectives and experiences of each group. One patient focus group included patient advocates (*n* = 5), and the other group discussion included patients (*n* = 2). At ACN, two groups of citizen advocates were formed, who were often also very knowledgeable or active in health-related advocacy. The first citizen advocate group (*n* = 8) consisted of a researcher, lawyer, administrator, pharmacist, physician, health manager and communication manager. The second citizen advocate group (*n* = 7) consisted of researchers, nurses, a nursing advocacy advocate and a director of a patient organisation. Additionally, five individual interviews were conducted with patients (*n* = 5) who were originally recruited through EPF, but were unable to attend the scheduled focus groups. To ensure their perspectives were still included, these participants were offered individual interviews as alternative. Additionally, one citizen advocate (*n* = 1), recruited via ACN was interviewed individually after being unable to attend the ACN focus group. The individual interviews lasted between 45 and 60 min. A citizen advocate was also included in the final feedback session after the completion of the interviews and focus groups to validate the findings and explore key areas in the study.

Upon completion, 29 participants took part in the study. The patients (*n* = 13) had experience with a variety of (hereditary and nonhereditary) disease areas, allowing the research to be broadly applicable rather than specific to one disease area.

Participants were recruited through EPF and ACN, representing active members of the public and patients’ community referred to here as “patients and patient advocates and citizen advocates”. While this description reflects advocates from patient and citizen organisations, individual patients varied in their levels of health literacy and advocacy experience.

The inclusion criteria for participation were an expressed interest in and prior experience with healthcare and preventive measures, ensuring that we gain insights from their knowledge while exploring new elements relevant for personalised prevention. In this study, a particular focus of personalised prevention was on genetic information. This approach allowed us to analyse the impact of genetic data on personalised prevention within the broader context of general healthcare, leveraging participants’ existing familiarity with prevention and care. We aimed to attain a range of perspectives across regions in Europe as well as the United Kingdom.

### Data collection and procedure

Both the interviews and focus groups were conducted online via the Microsoft Teams platform. Prior to their scheduled session, participants received an informed consent form, providing background information and definitions of key terms (Supplementary Material 2). This preparatory material enabled participants to be informed and engage meaningfully in the discussions. The recruitment and scheduling process was handled by the EPF or ACN to ensure a high level of confidentiality, giving the researchers as little access to participant information as possible prior to participation. The focus groups lasted between 90 and 120 min. The focus groups were facilitated by a moderator (CvE or LLK), observer/technical support (LLK), and a note-taker (KG or LLK) from the Amsterdam UMC, whereas a representative from the EPF or ACN, who recruited the participants, was present to welcome them.

PowerPoint slides were used during the focus groups to present key information. Following the first focus group, the introduction was simplified, and the sequence of the domains (Fig. 1) was streamlined to improve clarity and flow. For the individual interviews, no slides were used, but the introduction of personalised prevention and the questions per domain (Supplementary Material 1) were performed verbally. The focus groups and interviews were conducted between 24th April 2024 and 11th June 2024. No new themes were found and data collection was ceased.

### Feedback session

A feedback session was organised in July 2024 in collaboration with the EPF and ACN to gather input on the preliminary results of the study. Seven participants participated in the feedback session to validate the findings, including six previously interviewed or who participated focus groups (three citizens advocates from ACN, three patients or patient advocates from EPF) and one new citizen advocate from the PROPHET Stakeholder Forum [17], representing diverse groups of stakeholders: Ministry of Health employee, lawyer, researcher, physician, and patient advocates. They represented local, national, and European perspectives. The group comprised two males and five females.

Feedback was obtained through an online presentation of preliminary findings across Research, Care, and Governance, followed by structured discussion aimed to validate the findings and explore key areas, including identifying any missed areas in the study; distinguishing between the needs of patients and the public; suggesting additional tools and instruments to increase engagement and empowerment across diverse target groups; and clarifying the roles and responsibilities of professional organisations, governments, and other stakeholders. This feedback was instrumental in refining the study’s conclusions and recommendations.

### Data analysis

All the interviews were held online, audio recorded, and transcribed. The data were iteratively thematically analysed via MAXQDA software version Plus 2022 (Release 22.1.1) to ensure robust thematic coding and qualitative data organisation. The coding analysis was performed across the individual interviews and focus group transcripts. Initial coding of two interviews, comprising open coding and category identification, was performed by two researchers (LLK and KG) independently, which formed the basis for the analysis of the remaining interviews. Thereafter, a final codebook was developed and discussed with two researchers (LLK and CvE). On the basis of the codebook, the main themes and subthemes were identified and categorised across the Research, Care, and Governance domains by LLK and discussed with all the coauthors. Interviews and focus groups were conducted in English, the common language among participants from various countries, to facilitate clear communication.

## Results

### Participants

**Fig. 2:**
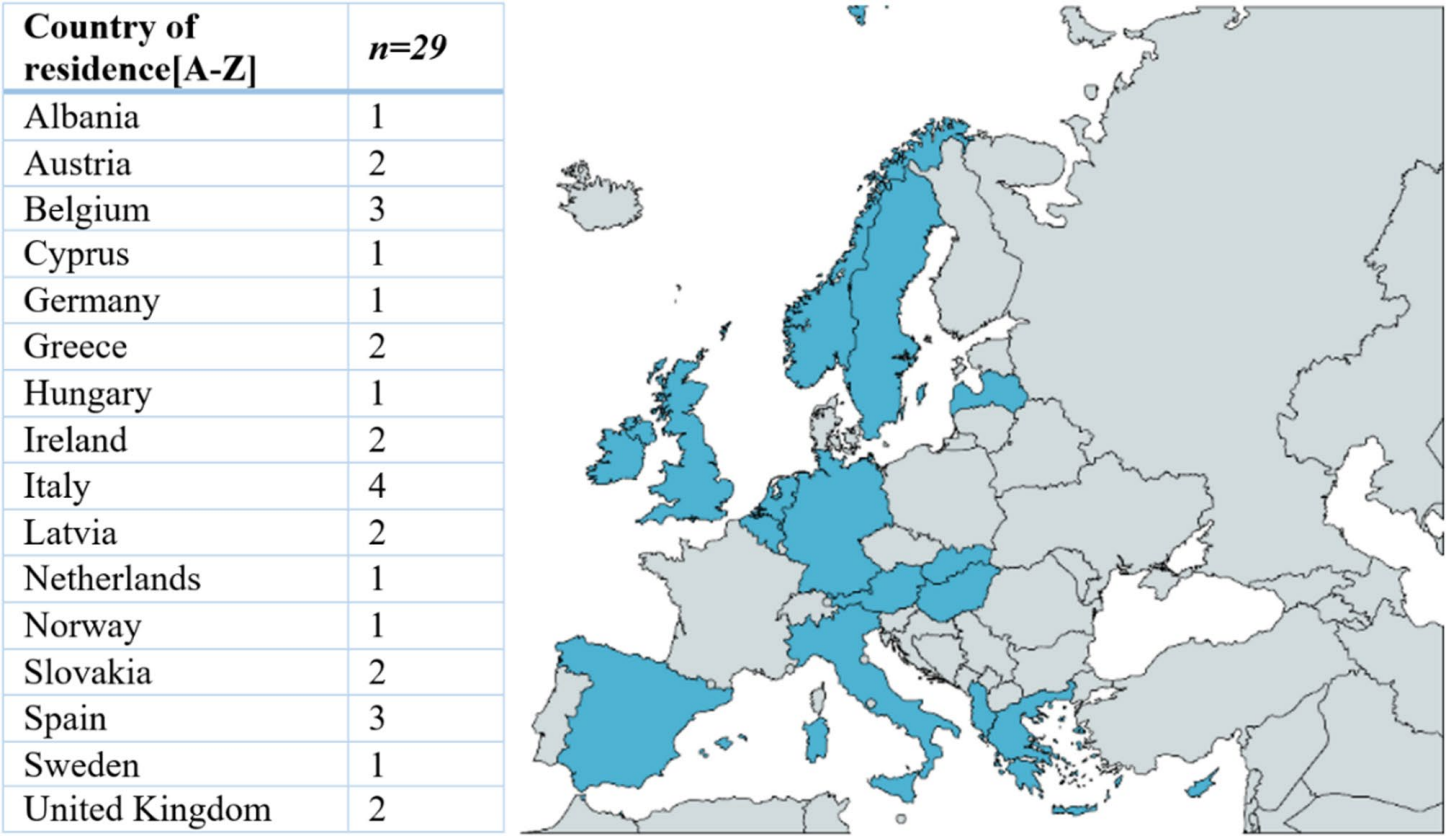
Representation of participants’ country of residence across Europe

The participants’ country of residence (*n* = 16) is shown in Fig. 2. In total, the sample included 12 patients and patient advocates and 17 citizen advocates. Among the patients, the conditions they reported were Parkinson’s disease (*n* = 4), cancer (*n* = 4), type 1 diabetes (*n* = 1), cardiovascular disease (*n* = 1), rheumatic disease (*n* = 1), and haemophilia/Von Willebrand disease (*n* = 1). Some citizen advocates were also involved in health advocacy or were personally affected by a disease but chose to contribute from the perspective of a citizen (advocate) or professional experience. Overall, the participants tended to be older and more highly educated. The majority of the participants were aged between 40 and 50 years (*n* = 12), followed closely by those aged 50 years and older (*n* = 10). The study sample consisted of 21 females and 8 males (see Table 1). The age and gender were assessed by the researchers.

**Table 1:** Demographic characteristics of the patients, patient advocates, and the citizen advocates

Participant characteristics	Active citizenship network (*n* = 16)	European patients forum (*n* = 12)	PROPHET stakeholder forum (*n* = 1) for feedback session	Total(*n* =29)
**Gender**				
Male	5	2 (1 patient, 1 patient advocate)	1	8
Female	11	10 (4 patients, 6 patient advocates)		21
**Age range**				
20–30				
30–40	3	1		4
40–50	9	3		12
50–65+	3	7		10
Unknown	1	1	1	3
**Professions of the citizen advocates** (*n* = 16)				
EU project manager	1			1
Director of non-profit organisation health related	1			1
Ministry of Health employee	2			2
Health policy expert	1			1
Lawyer and patient advocate	1			1
Nursing advocacy representative	1			1
Pharmacist	1			1
Medical doctor/physician	2		1	3
Professor (Primary Care, Population Health and Medicine)	2			2
Researcher	2			2
Administrative staff	1			1
Institute of Technology	1			1
**Disease areas of patients and patient advocates** (*n* = 12)				
Cardiovascular disease		1		
Breast cancer		3		
Cancer (general)		1		
Parkinson’s disease		4		
Haemophilia/Von willebrand disease		1		
Type 1 diabetes		1		
Rheumatic disease		1		

The participants were motivated to take part primarily by a desire to improve public and patient engagement in healthcare. Many participants were deeply involved in patient advocacy and healthcare organisations, driven by personal experiences with illness or professional roles in medicine, public health and patient safety, advocating for patient rights, and/or improving healthcare access and outcomes. The participants’ knowledge of personalised medicine and personalised prevention came from healthcare providers, conferences, and various media. However, understanding varied, with some participants having detailed insights and others being uncertain about practical applications.

### Themes

The findings are clustered across three key themes within the domains of Research, Care, and Governance, offering a comprehensive understanding of the various facets of public and patient engagement in personalised prevention: (i) Information and Communication; (ii) Representation and Inclusivity; and (iii) Ethical and Regulatory Considerations.

#### Research domain

The findings reflect participants’ views on enhancing patient and broader public engagement and empowerment in personalised prevention research practices across the three main themes, as displayed in Table 2. The need for clear *Information and Communication*, specifically regarding the information to resonate with people’s experiences while respecting their level of understanding, was highlighted by participants (Research Quote RQ01). The participants mentioned the need for open communication and involvement at every stage of the research process, including shaping research questions, to feel truly engaged in personalised prevention (RQ02). In terms of *Representation and Inclusivity*, the participants emphasised the needs of diverse groups in research to develop equitable strategies (RQ03), specifically regarding data collection, owing to concerns about the lack of representation (RQ04). The participants echoed that marginalised groups are overlooked. Engaging these communities ensures that their concerns and needs are included (RQ05). With respect to *Ethical and Regulatory Considerations*, participants advocated compensating their time when they participated in research as a matter of fair compensation and recognition of their time and contribution (RQ06).

**Table 2:** Themes, subthemes, exemplar quotes and interpretations across the research domain

Research domain			
Main theme	Subtheme	Quote	Research Quote (RQ) #
Information and communication	Clarity in language	*“I believe that it [communication] works best when the language is understandable to the general public and close to their real-life experience.” (public#07_FG)*	RQ01
	Participation in research	*“It is important there is […] open communication and that the community and the citizens feel involved at every step of the study*,* including identifying the research questions” (public#16_FG)*	RQ02
Representation and inclusivity	Diverse Representation	*“We are starting to address inequalities in public health. We are focusing on vulnerable populations including people in prisons*,* immigrants*,* those living alone*,* and even illegal immigrants in our region. Our efforts […] aimed at economic ethics and chronic diseases*,* particularly focusing on methodologies that are cost-effective and sustainable within the healthcare system.” (public#28_interview)*	RQ03
	Representation in data collection	“*We never collect data for non-white populations. […] the results not involving different ethnic backgrounds in research means that actually the various treatments […]are not actually fit for purpose.” (public#07_FG)*	RQ04
	Marginalised groups	*“I think it is really important to work with marginalised groups to make sure that they*,* their concerns and their fears or their thoughts in general are addressed and included in the research because of representation. And I think they’re often overlooked*,* especially if they’re hard to reach.” (public#14_FG)*	RQ05
Ethical and regulatory considerations	Equitable participation	*“But [what] I find often helpful or also fair is to use incentives or compensation to give people something for the time that they spend on the research. […] I think it is […] a matter of respect. […] we should remember [to] reimburse them for their time.” (public#14_FG)*	RQ06

#### Care domain

In the Care domain, several subthemes emerged, as depicted in Table 3. Within the theme of *Information and Communication*, the results show that effective, comprehensive communication with the citizen advocates and patients remains a key challenge, particularly in regard to personalised prevention. Tailoring messages concerning individual health needs, risks, and circumstances is essential for improving engagement and encouraging proactive health behaviour. Despite several participants having firsthand experience with genetic testing, gaps in knowledge about how personalised prevention could impact their overall health care were evident, as they also noted that they did not think they received sufficient information to grasp implications of the genetic test results (Care Quote CQ01). Tailoring communication to different age groups was noted, stressing the need for multiple channels to ensure effective communication (CQ02). Patients mentioned finding doctors’ replies too complex, possibly resulting in patient advocates having to take additional steps to make sense of the information and highlighting the need for clearer, comprehensive information in lay language (CQ03).

Several participants indicated a need for a holistic care approach that transcends traditional approaches. This included not only emphasising the physical health of individuals but also integrating their emotional, mental, and social well-being (CQ04), as well as balancing individual health data with predictive models that incorporate broader environmental and cultural factors (CQ05), to better inform personalised prevention strategies.

Under the umbrella theme of *Representation and Inclusivity*, participants emphasised the necessity of prevention due to fundamental healthcare challenges, including healthcare staff shortages. Having nonspecialist mentors who offer support and share practical experiences can be valuable for patients, providing them access to shared knowledge in a more informal setting (CQ06). Furthermore, concerns about the broader implications of personalised prevention for healthcare systems and social equity were mentioned due to a potential shift from a solidarity-based healthcare system to one focused on individual risk (CQ07). Quote CQ08 highlights the importance of considering the broader social context of individuals and the potential role of local communities and authorities in primary prevention efforts, especially for populations that may be difficult to reach through traditional healthcare channels.

*With respect to Ethical and Regulatory* considerations within the Care domain, participants’ concerns about how genetic information could be utilised by insurance providers, potentially impacting access to health insurance benefits, were reiterated by patients (CQ09). Moreover, the lack of access to their medical records was mentioned, indicating a desire for greater control over their own data. Patients emphasised the importance of being able to view and manage their health information to facilitate informed participation in their treatment (CQ10).

**Table 3:** Themes, subthemes, exemplar quotes and interpretations across the care domain

Care domain			
Main theme	Subtheme	Quote	Care Quote (CQ) #
Information and communication	Prevention and genetic test	*“I went through the process of doing genetic testing for my cancer; the doctor gave me much more information [about the test]*,* not so much about prevention. How can personalised medicine help me depending on the results of the genetic testing and how might the checkups or the treatment plans change in the future based on the genetic results? […] I hadn’t received enough information about what this means.” (patient#04_interview)*	CQ01
	Use of multiple channels	*“We work with different ways of disseminating information through various channels. For young people*,* social media works more [better]*,* but for older people*,* face-to-face interactions or advice in primary care centres work better. It is about using a combination of channels to communicate effectively with different groups.”* (*public#*28_interview)	CQ02
	Bridging communication gap	*“The replies of the doctors are very scientific and very*,* very*,* difficult to understand. And so*,* we are […] reworking these replies*,* making them [understandable] in the most […] human way*,* and afterwards we are sending [them] back to the doctor for the approval that we reworked his answers correctly” (patient#21_interview)*	CQ03
Representation and inclusivity	Person in personalised prevention	*“When I realised how life-altering the results can be*,* I needed mental preparation and time […] despite initially agreeing to genetic testing for breast cancer*,* the complexity of the potential outcomes became evident during the waiting period*,* which coincided with my chemotherapy and exacerbated my mental health challenges. It’s important for healthcare professionals to communicate on mental health [*…*] to take an extra step beyond the treatment plan. I think most of the points we made come down to more personalised medicine […] seeing the person as a whole would really change the experience in the hospital…being able to read what you sign and the information linked to the treatment*,* that is the bare minimum of inclusion I think.”* (patient#04_interview)	CQ04
	Integrated care	*“We need to think in a more holistic approach including social environments*,* municipalities*,* culture*,* and other sociodeterminants of health. We need to balance individual decisions*,* with individual data*,* but we also need to put in the predictive models based on environments and culture and other data that is not individual.” (public#28_interview)*	CQ05
	Community engagement	*“It’s important for patients to have a mentor—someone not necessarily a specialist*,* but willing to share experiences and support the patient. This helps patients realise they are not alone and can share lifehacks and information anytime*,* through a chat or community.” (patient#21_interview)*	CQ06
	Shift of healthcare system	*“What we [have seen is] that healthcare systems with healthcare insurance systems in Europe are traditionally based on solidarity. And I’m wondering whether we are moving away from this solidarity principle and going rather to the individual risk calculations […] I hope this will never happen in Europe […].” (patient#23_FG)*	CQ07
	Inclusivity	*“We need to think that people are in the community with other people […] and not only with health professionals […]. It is important to reach citizens*,* immigrants*,* and all people*,* focusing on primary prevention by working with municipalities and reaching out to those who are hard to reach.” (public#28_interview)*	CQ08
Ethical and regulatory considerations	Exclusion: health insurance	*“Privacy*,* particularly in terms of insurance providers*,* and […] the possibility of that information being used as a tool to exclude someone from health insurance benefit [is concerning].” (patient#03_FG)*	CQ09
	Access to medical data	*“I cannot see my file on the network [health portal]. My son wanted to consult another doctor specialist and he asked this clinic to transfer the file. They never did. It is important that persons involved in the treatment [are] informed.” (patient#05_interview)*	CQ10

#### Governance domain

As depicted in Table 4, the participants emphasised the importance of public and patient engagement in the Governance domain of personalised prevention. In the theme of *Information and Communication*, patients highlighted the necessity of regular, structured engagement opportunities where patient advocates can participate in discussions with policymakers and healthcare authorities (Governance Quote GQ01). Trustworthy and evidence-based information at the national and European levels was mentioned to support patients, patient organisations and the wider public in engaging with personalised prevention (GQ02).

Regarding *Representation and Inclusivity*, participants stressed the importance of inclusive governance and policymaking to address diverse populations in personalised prevention. A call for “health democracy” reflects the need for patient voices to be recognised and included in decision-making processes, reinforcing that healthcare quality impacts everyone, as anyone can become a patient (GQ03) while at the same time ensuring that patients’ specific needs are reflected in policies. Initiatives aimed at cultural sensitivity were particularly highlighted (GQ04). Various patients and citizen advocates mentioned examples of engagement initiatives, such as mentor chats, patient-focused newsletters/magazines and patient involvement and feedback in curriculum development for medical students. The participants argued that governments should ensure proactive healthcare, criticising the system for being reactive despite abundant available data (GQ05).

With respect to *Ethical and Regulatory considerations*, the importance of data quality and protection, along with the need for responsible data sharing, was emphasised. The participants support quality assurance for integrating data from various sources into health systems, including wearables (GQ06). They emphasise partnerships with the broader public and better use of patient-generated data, stressing that healthcare data should be utilised by governments as a tool for disease prevention and the development of predictive models (GQ06). Issues were raised about private companies managing health information (GQ06). The general acceptance of data sharing to enhance health outcomes was echoed by many respondents (GQ07). A participant noted as a principle for institutions is crucial for building public trust, especially when implementing national policies on privacy and data sharing within personalised healthcare settings (GQ08).

Moreover, several issues have been raised regarding affordable patient and public access to prevention. While many devices aimed at disease prevention are beneficial, participants noted the “unjustifiable” high costs of preventive devices, which they argued create inequities in access (GQ09). They emphasised that it is the government’s responsibility to ensure that these devices are widely accessible, as private companies may not prioritise prevention (GQ09).

**Table 4:** Themes, subthemes, exemplar quotes and interpretations across the governance domain

Governance domain			
Main theme	Subtheme	Quote	Governance Quote (GQ) #
Information and communication	Structural Engagement and Regular Feedback	*“And how to best engage patients? Invite the advocates of the patient organisations to the meeting*,* with the authorities*,* the Parliament*,* local committees or maybe with the Health Minister*,* have round tables and discussions*,* and listen […] what is important is to make these discussions regular*,* so with a follow up.” (patient#21_interview)*	GQ01
	Quality evidence-based information	*“[…] I think what we need on the national and even on the European level is a high-level platform with quality evidence-based information that patients*,* citizens and patient organisations can trust.” (public#12_FG)*	GQ02
Representation and inclusivity	Health Democracy	*“It should be more and more about health democracy; patient communities really need to be seen and to be heard. And when we do it together*,* I really think we can see results. Everybody could be a patient at some point. So the quality of healthcare really affects all of us.” (patient#26_FG)*	GQ03
	Diverse Representation	*“Diversity and equity are central to effective personalised prevention. It is essential to consider cultural nuances and adapt healthcare practices accordingly.” (patient#21_interview)*	GQ04
	Healthcare system is reactive and not proactive	*“I think it is the obligation of the governments who organise the healthcare system to take care of patients’ health status. The healthcare system is reactive and not proactive. There are a lot of data available in the healthcare system and outside […] We should channel this information into healthcare.” (public#10_FG)*	GQ05
Ethical and regulatory considerations	Feeding information into health systems	*“I agree there should be a quality assurance system for feeding information into health systems from various sources outside of health systems. […] I think healthcare data should be a means for the government to prevent diseases*,* to build prediction models. There should be a partnership with citizens because there are a lot of means through which patients generate data about their own healthcare like smartwatches. […]Private companies should also have the responsibility and should allow citizens to transfer the information they collect to national health systems. This data could be a valuable source for the whole healthcare system*,* but it is not explored.” (public#10_FG)*	GQ06
	Sharing data	*“I don’t have much against sharing my own data*,* if they can help […] I will do it [share my data].” (patient#27_interview)*	GQ07
	Need for regulation	*“Regarding the privacy data sharing and trust point*,* I think there we can also add trust/transparency because I think it is very important for when […] national policies are implemented for privacy and data sharing*,* that there is transparency from the institutions. How will this be used?” (public#20_FG)*	GQ08
	Unjustifiable prices for prevention devices	*“If we focus on the prevention part*,* there are millions of devices that focus on prevention which are quite helpful. The price is not justifiable*,* it is very high. […] It is the role of the government to make those devices massively available […]*,* private companies don’t care about prevention*,* so the State responsibility first of all is working on prevention.” (public#29_FG)*	GQ09

## Discussion

The findings highlight several key elements for enhancing patient and broader public engagement in personalised prevention initiatives according to patients and citizen advocates, thereby increasing empowerment, strengthening acceptance of personalised prevention approaches and improving their implementation. To increase empowerment across the domains of Research, Care, and Governance, clear, accessible healthcare information is vital, particularly regarding advancements such as integrating genetic information into personalised prevention strategies.

Throughout the focus groups and interviews, concerns about equity were raised across all three domains, which calls for a reflection on how to integrate equity considerations into the implementation and governance of personalised prevention approaches from the outset. Diverse representation in research emerged as a critical factor, with participants stressing the need to include marginalised and underrepresented groups to avoid biased outcomes and ensure that research reflects the needs of all populations. There is increased recognition that improving research inclusiveness enhances the accuracy of and trust in research findings, contributing to more equitable health outcomes [18, 19]. This aligns with ongoing initiatives by regulatory agencies to promote diversity and inclusion in clinical trials, although efforts must be strengthened across jurisdictions [20, 21]. The high cost of preventive devices, such as wearables and genetic tests, was also flagged in the interviews as a barrier to equitable access. The participants called for government intervention to improve affordability. This concern resonates with the work of Gomathi and Mishra [22] and Pang et al. [23], who emphasise the need to address accessibility and inclusivity in the digital transformation of healthcare. This calls for specific models to finance healthcare systems’ digital transition and prevention-based approaches.

In the Research domain, the participants emphasised that scientific findings and data must be conveyed in a manner that is clear and understandable to nonexperts and resonates with personal experiences. As stated by Woolf et al., authentic engagement encompasses involving patient and broader public stakeholders as full partners in all phases of research [24]. Meaningful patient participation in research, including designing research priorities and assessing results, depends on clear information and communication with patients. Some recent projects have focused on developing tools to guide researchers on how to better involve patients and training programs for patients [25].

In the Care domain, challenges in effectively communicating complex genetic concepts and tailoring information to individual patients have been identified. These challenges underscore the importance of equipping healthcare professionals with the necessary knowledge and skills to close their own knowledge gaps, so they are equipped to support patients by addressing patients’ misunderstanding and information needs. Insufficient information may impede patients’ understanding, potentially undermining their trust in healthcare providers and reducing their adherence to personalised prevention strategies. Such gaps can ultimately hinder patients’ ability to make informed health decisions, highlighting the need for improved communication and support in personalised care. This indicates the importance of quality genomics (online) education for (genetic) healthcare professionals. To achieve more comprehensive and personalised health management, enhancing the digital health literacy of both patients and healthcare professionals is crucial, leading to better integration of nonclinical assessments into patient care [26].

The concept of patient-centred care was prominent in the Care domain, with participants favouring healthcare practices that prioritise individual patient preferences, values, and needs. According to the literature, patients with chronic illnesses who actively participate in care appear to survive longer than those who do not participate yet receive comparable care [27, 28]. While patients often focus on care and secondary or tertiary prevention, the citizen advocates may be primarily concerned with primary prevention, highlighting the distinct yet complementary needs within the domain. The participants value providers who listen, understand their concerns, and involve them in decision-making, highlighting the need for an integrative approach [29] that addresses physical and mental health. Beyond their individual care and prevention, enabling patients and the public to actively contribute to policy discussions through advisory groups, expert panels, and public consultations ensures that their insights inform preventive strategies [30]. Moreover, follow-up mechanisms are needed to ensure that patient and public inputs are acted upon.

The role of personalised prevention in the evolution of the healthcare system emerged as a key aspect for participants. There is broad support for a shift toward preventive care and a shift away from systems focused mostly on disease and treatment, with participants viewing prevention as a way to improve the sustainability of healthcare systems. Research supports this shift from reactive to proactive healthcare, optimising societal returns and benefiting a larger population [31]. However, concerns have been raised about personalised prevention, potentially moving away from solidarity-based healthcare systems towards individual risk assessments. This underscores the need for robust regulatory frameworks to balance individual care needs with collective well-being.

Within the Governance domain, safeguarding data privacy has emerged as a key concern, with participants advocating for clearer policies on how health data are used, shared, and protected. This mirrors ongoing debates in the European Health Data Space Regulation, which aims to promote the public’s control over their health data while facilitating data sharing [32]. Similarly, for digital tools such as wearable devices, regulatory frameworks need to guarantee accuracy and protect patient data privacy to promote trust in personalised health monitoring while keeping these technologies affordable. Public trust in governments’ management of health data, as opposed to private companies, calls for better integration of these data into efficient national health systems [33]. This, in turn, places pressure on governments to invest in digitalisation and secure infrastructure.

There is increased recognition in policy debates of the value of incorporating the patient perspective to ensure that healthcare services better meet the needs of those they aim to serve. The Organisation for Economic Cooperation and Development (OECD) launched Patient-Reported Indicator Surveys (PaRIS) to create a new generation of indicators that measure healthcare outcomes and experiences most relevant to patients, promoting more people-centred primary care [34]. However, improving patient participation in prevention requires overcoming challenges such as institutionalising patient organisations, ensuring fair compensation for participation, and significant efforts to increase communication, (digital) health literacy, and training.

The concept of “health democracy” resonated, with participants advocating for the recognition of patient communities as integral partners in shaping healthcare. There was strong support for community-based approaches to personalised prevention, extending beyond individual care to include the role of support networks, patient advocacy groups, and community engagement initiatives.

### Points to consider

Based on our findings, we suggest the following key points to consider across the Research, Care, and Governance domains regarding the engagement of patients and citizen advocates in personalised prevention, as displayed in Table 5.

**Table 5:**
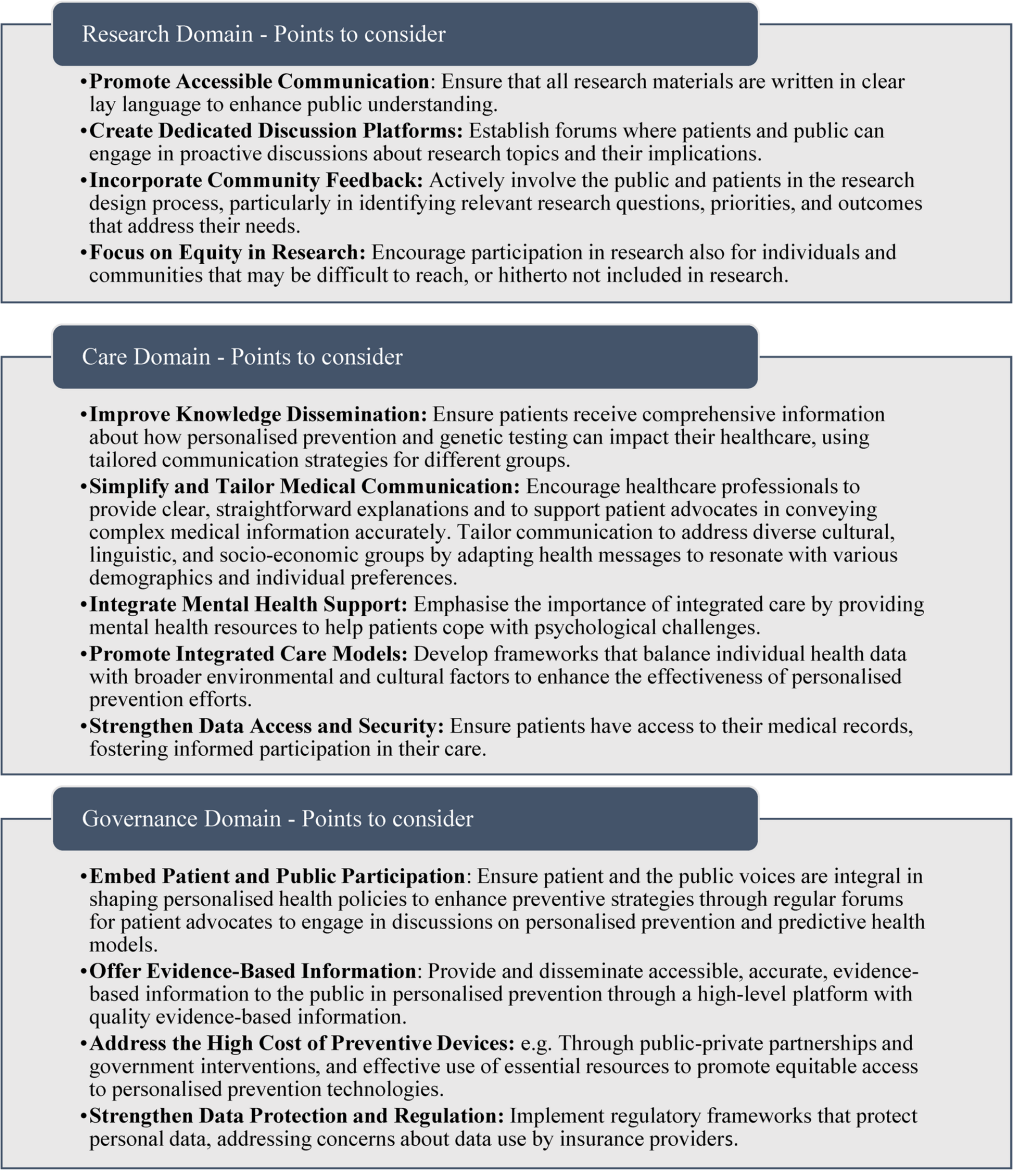
Points to consider: based on the study findings across the three domains (Research, Care and Governance) regarding the engagement of patients and the public in personalised prevention

### Strengths and limitations

The strengths of this study include the rich qualitative data obtained through semi-structured interviews and focus groups, providing nuanced insights into public and patient engagement in personalised prevention. The diverse participant pool encompasses various stakeholders across multiple European countries. The inclusion of participants from different disease areas further enriched the perspectives gathered. The active involvement of patient advocates, who are well versed in health policy and advocacy, added depth to the analysis. This study has several limitations. First, the analysis was restricted to a limited number of diseases, which may affect the broader applicability of the findings. Additionally, the sample size was small in each country, which could introduce variability in the results. Moreover, the study involved countries with diverse healthcare systems, which may have contributed to differences in outcomes and hindered the ability to draw universally applicable conclusions. Sampling bias towards those already engaged in healthcare, potentially excluding less engaged or marginalised populations or the public and patients who are not members of a patient or public organisation, may have affected the results. While online interviews and focus groups allowed broader geographical participation and comfort for patients, they excluded those lacking technological/digital literacy, and nonverbal cues may have been missed. Additionally, personalised prevention was sometimes interpreted as lifestyle changes rather than the integration of wider data, such as genetic information or biomarkers, possibly affecting findings. The demographic data were skewed towards older, higher-educated participants. The use of English, a nonnative language for most, may have limited the full expression of thoughts despite efforts to clarify misunderstandings.

## Conclusion

While personalised prevention has the potential to improve health outcomes significantly, its implementation in healthcare practice raises significant challenges, from limited awareness to a lack of infrastructure and insufficient healthcare professionals’ training, and acceptance of patient and broader public engagement and empowerment in healthcare. Most importantly, patient and public engagement in personalised prevention is essential for the successful implementation of personalised prevention strategies: the concept itself relies on health literacy, proactive engagement of the public with the healthcare system, health data sharing, and an enhanced patient‒doctor relationship.

This study has shown that promoting patient and public engagement in personalised prevention requires a strategic focus across the Research, Care, and Governance domains. In each domain, addressing patients’ and public concerns related to *Information and Communication*,* Representation and Inclusivity*, and *Ethical and Regulatory Considerations* is vital to gaining public support for personalised prevention strategies. This involves, in particular, the availability of clear and accessible information, community-based initiatives to reach the wider public, including the most vulnerable, and a regulatory framework that promotes trust and transparency. Engaging patients and the wider public in the development and implementation of initiatives and policies to address these issues will facilitate the integration of personalised prevention into healthcare practice. Most importantly, however, it ensures that personalised prevention achieves public health objectives and effectively contributes to more equitable and resilient healthcare systems.

## Supplementary Information

Supplementary Material 1.

Supplementary Material 2.

## Data Availability

The datasets (transcripts) generated and/or analysed during the current study are not publicly available for privacy reasons. However, anonymised interview transcripts and a description of the coding trees are available from the first author upon reasonable request.
