# Supplementary material for: Personalised prevention: what patients and citizen advocates want for better engagement – a qualitative study
Source: BMC Public Health. 2025 Nov 4;25:3777. doi: 10.1186/s12889-025-24925-0 (PMC12584456; doi:10.1186/s12889-025-24925-0)
Supplement: Supplementary file 1 — Supplementary Material 1. [file 12889_2025_24925_MOESM1_ESM.pdf]

Supplementary Material 1

Interview guides used in the study by Kreeftenberg et al. Personalised Prevention: What Patients and Citizen Advocates Want for Better Engagement – a Qualitative Study

Interview guide: Patients

Welcome & Introduction: I would like to start with introducing myself and our team (mention names, institutions) and PROPHET briefly.

We would like to record the meeting for our reporting, if you agree we will now start the recording.

Could you please mention:

- Name?
- Where you are from/Country of residence?
- Do you represent an organisation?
- Motivation to participate?

Brief explanation of PROPHET project:

Develop research and innovation agenda for personalised prevention, with many stakeholders, such as patient organisations, universities.

And our task is to study patient and citizen engagement, so we do this as the Amsterdam University medical center together with patient representatives EPF and ACN.

Personalised medicine aims to tailor health care and prevention more to individual needs and characteristics, away from the one size fits all approach. Many definitions exist, but the core is to also include genetic information, in addition to other types of data on lifestyle, and also individual preferences or values.

Ideally **patients would play an active role** in their own health management, so they would need to understand that genetic information is available and discuss health care options with their health care providers, e.g. via shared decision making, potentially contribute health data to further research.

Summary of Purpose:

As we described in the information package we are conducting this interview to hear your perspective on personalised prevention. We are particularly interested in your views on how patients and the public can be better engaged and empowered in this area.

Your input will contribute to developing best practices for patient engagement in personalised prevention.

House Rules

- Checking consent form, confidentiality - will treat the information confidentially, we will not use names in the reports.
- Have you had a chance to go through the handout?
- You will be asked a series of questions for discussion
- Please feel you can speak openly and honestly
- No right or wrong answers – we are simply interested in your experiences and opinions

Do you have any questions at this point?

Interview Overview:

During the interview, we'll be asking you a series of questions covering three main areas: Research, Care, and Governance. They all relate to how best to engage citizens in personalised prevention/medicine

Questions per Domain

Care domain: How can patients be empowered and educated?

Your lessons learned from personalised prevention/personalised medicine:<br>• What tool worked best, why?<br>• How can this be improved?<br>• What is missing?
Do you have an example of effective communication tools? Or what could be done to improve communication by healthcare professionals (easy to understand content, toolkits, meetings, etc.)?
Is there a difference between patient or citizen representation?
How can patients best be involved in governance of care in personalised prevention?
Do you know of patients who were offered a genetic test as part of their care pathway, how did they feel about that?<br>Carefully probe:<br>- would you have wanted to know before<br>- would it have changed your lifestyle, if prevention is possible
**Research domain: how to improve patients involvement?**
Your lessons learned personalised prevention/personalised medicine:<br>• What tool worked best, why?<br>• How can this be improved?<br>• What is missing?
How best to communicate research findings/ research to patients:
How can patients best be involved in governance of research in personalised prevention?<br>• What worked best, why?<br>• How to ensure diverse representation?
If not addressed yet:<br>• Do you feel comfortable participating in research (check: sharing your health data)<br>• Do you feel comfortable participating in genetic research (check: sharing genetic data)
**Wrap-up question**
What are the two or three most important things that you think need attention?
Do you want to be informed of the results? How?
Thank you for your participation!

**Interview guide: Citizen Advocates**
Welcome & Introduction: I would like to start with introducing myself and our team (mention names, institutions) and PROPHET briefly.
We would like to record the meeting for our reporting if you agree we will now start the recording
Could you please mention:
• Name?<br>• Where you are from/Country of residence?<br>• Do you represent an organisation?<br>• Motivation to participate?
**Brief explanation of PROPHET project:**
Develop research and innovation agenda for personalised prevention, with many stakeholders, such as patient organisations, universities.
And our task is to study patient and citizen engagement, so we do this as the Amsterdam University medical center together with patient and citizen representatives EPF and ACN.

**Personalised medicine** aims to tailor health care and prevention more to individual needs and characteristics, away from the one size fits all approach. Many definitions exist, but the core is to also include genetic information, in addition to other types of data on lifestyle, and also individual preferences or values.
Ideally **patients would play an active role** in their own health management, so would need to understand that genetic information is available and discuss health care options with their health care providers, e.g. via shared decision making, potentially contribute health data to further research.
**Summary of Purpose:**
As we described in the information package we are conducting this interview to hear your perspective on personalised prevention. We are particularly interested in your views on how patients and the public can be better engaged and empowered in this area.
Your input will contribute to developing best practices for patient engagement in personalised prevention.
**House Rules**
• Checking consent form, confidentiality - will treat the information confidentially, we will not use names in the reports.<br>• Have you had a chance to go through the handout?<br>• You will be asked a series of questions for discussion<br>• Please feel you can speak openly and honestly<br>• No right or wrong answers – we're simply interested in your experiences and opinions
Do you have any questions at this point?
**Interview Overview**:
During the interview, we'll be asking you a series of questions covering three main areas: Research, Care, and Governance. They all relate to how best to engage citizens in personalised prevention/medicine
Questions per Domain
**Care domain: How can citizens be better educated and empowered**?
Your lessons learned from personalised prevention/personalised medicine:<br>• What tool worked best, why?<br>• How can this be improved?<br>• What is missing?
How to empower and educate citizens to be prepared for personalised prevention
Is there a difference between patient or citizen representation?
How can citizens best be involved in governance of care in personalised prevention?<br>• What worked best, why?<br>• How to ensure diverse representation?
**Research domain: How are citizens involved?**
Your lessons learned:<br>How best to involve citizens on setting e.g. research topics, priorities, design etc.
Can you describe one example that <u>worked well</u>, addressing e.g.:<br>• What tool worked best, why?<br>• How can this be improved?
How best to communicate about research to citizens:<br>• What tools worked best, why?<br>• Is information understandable / accessible?
How can citizens best be involved in governance of research in personalised prevention?<br>• What worked best, why?<br>• How to ensure diverse representation?
**Wrap-up question**

What are the two most important things that you think need attention in empowering citizens in personalised prevention/personalised medicine?
Do you want to be informed of the results? How?
Thank you for your participation!
