# Supplementary material for: Personalised prevention: what patients and citizen advocates want for better engagement – a qualitative study
Source: BMC Public Health. 2025 Nov 4;25:3777. doi: 10.1186/s12889-025-24925-0 (PMC12584456; doi:10.1186/s12889-025-24925-0)
Supplement: Supplementary file 2 — Supplementary Material 2. [file 12889_2025_24925_MOESM2_ESM.pdf]

Supplementary Material 2
Participant Handout used in the study by Kreeftenberg et al.
Personalised Prevention: What Patients and Citizen Advocates Want for Better Engagement – A Qualitative Study

Participant Handout: PROPHET Personalised Prevention Discussion

Defining guidelines and best models for citizen and patient engagement

Thank you in advance for your time and participation in this study about your experiences and thoughts on citizen and patient engagement in personalised prevention. This handout will provide some background information on the topics we will discuss together.

Purpose of this discussion

The purpose of this discussion is to help develop better guidelines for engaging citizens and patients in implementing personalised prevention throughout the European Union, to ensure that their needs and concerns are reflected.

We encourage you to think about and reflect on your personal experiences relating to personalised medicine and personalised prevention ahead of the meeting. Given your experiences, we hope to ask about how to communicate about personalised prevention, barriers to using personalised prevention, and ideas for improvements in citizen and patient engagement. We will also ask you about (ethical) concerns you may have.

List of terms

Here are a few concepts we will use during the discussion:

- **Genomics**: the study of all of a person's genes, including the interaction of those genes with each other and with the environment[i]
- **Personalised Medicine**: Personalised medicine (PM) is a form of medicine where a doctor uses information about the person's genetic profile and other data to guide decision-making about prevention, diagnosis and treatment. The idea is that you tailor "the right therapeutic strategy for the right person, at the right time." [ii],[iii],[iv]

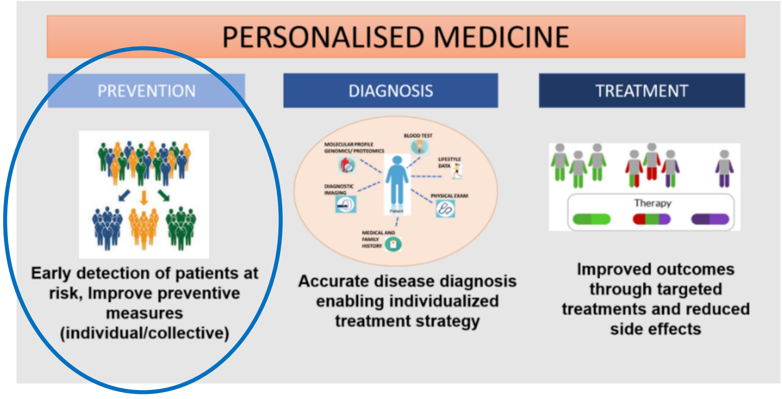

- **Personalised Prevention:** Personalised prevention is a form of PM. "Personalised prevention aims to prevent onset, progression and recurrence of diseases through the adoption of targeted interventions that consider the biological information*, environmental and behavioral

characteristics, socio-economic and cultural context of individuals. This should be timely, effective and equitable in order to maintain the best possible balance in lifetime health trajectory”[v]

- * e.g. genetic and other biomarkers, demographics, health conditions

How do personalised medicine and personalised prevention connect? Every person has a unique health history. More concretely, personalised medicine and prevention come together to give tailored treatment and prevention recommendations based on a person’s genetics *as well as* their overall health history and environment. For example, breast cancer can be caused by many different genes, environmental influences, demographics, and other previous health conditions[vi]. While breast cancer screening is generally offered based on age for the general population, families with higher genetic risks are seen in the clinic. Screening can be adapted (personalised), for instance start at an earlier age or be done more frequently, depending on the risk group.

- **Engagement**: There are many ways to define engagement, but for our discussion, we use engagement to mean:
  - At the Individual level: “the extent to which patients, [citizens] and their families or caregivers, whenever appropriate, participate in decisions related to their condition (e.g. through shared decision-making, self management) and contribute to organizational learning through their specific experience”
  - At the Collective level: “the extent to which patients [and citizens], through their representative organizations, contribute to shaping the health care system through involvement in health care policy-making, organization, design and delivery.”[vii]
  - Why engagement? Engagement is important because it gives citizens and patients a voice in naming their concerns, priorities, and desires. The objective of engagement is to ensure that healthcare and health care policies meet the needs of those who use and receive these services, namely patients and the public. Engagement goes hand in hand with access to information, which is a key aspect of patients’ and citizens’ empowerment.

- **Empowerment**: Like engagement, empowerment has many meanings. In our discussion, we refer to empowerment as a: “process that helps people gain control over their own lives and increases their capacity to act on issues that they themselves define as important.”[viii]

- **ELSI**: abbreviation for Ethical, Legal and Social Implications (in our discussion, the issues and concerns you may have about personalised medicine and personalised prevention)

Explanation of Domains

During our discussion, we will explore three domains related to the implementation of personalised prevention, namely: research, care, and governance/policy-making. Below are some explanations with sub-questions about the different domains. In each of these domains, patients and citizens can be involved in personalised prevention practices in different ways. There are overlaps between these domains so we understand if you may see connections and/or similarities between these three domains.

Research: Involvement of patients and citizens in research decision making, e.g. setting research agenda priorities, and communicating research to the public

- ➢ How best to involve patients and citizens?
  - Examples of what works, what can be improved?
    - E.g., engagement tools, level of involvement (high/low), barriers to engagement
  - Improving communication about research

Care: Increase the public and patients' active participation in healthcare. This includes: communicating risks, such as genetic test results, improving health literacy.

- How best to educate and empower patients and citizens in healthcare?
  - Examples of what works, what can be improved?
    - E.g., educational tools, level of involvement (high/low), barriers to empowerment

Governance: Participatory decision making in health policy (having a seat at the table)

- How to establish trust?
  - Examples of what works, what can be improved?
    - E.g., engagement tools, level of involvement (high/low), barriers to engagement
  - How to make sure all voices are heard?

ELSI issues

Towards the end of our discussion, we want to ask you about the concerns you may have about implementing personalised prevention. We will do a ranking exercise and give you some time to reflect on your responses. Here are some examples of ELSI issues that have arisen in recent literature[ix] and that we would like to discuss with you:

- Privacy, data sharing and trust: e.g. who has access to health records, concerns about data leaks, trusting health care professionals and the government in handling and storing their data safely
- Discrimination: e.g. genetic discrimination, insurance coverage limitations if you have a genetic predisposition (higher chance of developing) to a health condition
- Physician-patient relationship: e.g. lack of time during consults, training of healthcare professionals to use personalised prevention
- Dealing with uncertainties, becoming a patient-in-waiting: e.g. The mental and emotional stress of dealing with uncertain test results, knowledge about health problems that might arise in the future, etc.
- Overtreatment: the possibility of overtreatment based on preliminary testing
- Equity: We know more about genomes and disease-causing genes from people of European descent. Not all disease-causing genes are the same in different populations. How can we make personalised prevention relevant for all populations?
- Cost & access to healthcare: whether or not health insurance covers personalised prevention, potentially higher costs of testing and healthcare for the patients and health system
- Access to information technologies: e.g. digital literacy, using/accessing electronic health records

PROPHET Project

This research is part of the PROPHET project: a Personalised Prevention roadmap for the future HEalThcare. The PROPHET project is an European wide collaboration to create a roadmap, together with input from a broad range of stakeholders, including citizens, patients, healthcare professionals, and policy makers, to help implement personalised prevention.

More information about the PROPHET project can be found at this link: Home - PROPHET (prophetproject.eu)

[i] Source: https://www.genome.gov/about-genomics/fact-sheets/A-Brief-Guide-to-Genomics
[ii] Source: https://www.genome.gov/genetics-glossary/Personalised-Medicine
[iii] Source: Personalised medicine - European Commission (europa.eu)
[iv] Image source: What is Personalised Medicine? (eulac-permed.eu)
[v] Source: Methodology section - PROPHET (prophetproject.eu)
[vi] Source: Breast Cancer Risk Factors You Can't Change | American Cancer Society
[vii] Source: https://www.eu-patient.eu/globalassets/campaign-patient-empowerment/epf_briefing_patientempowerment_2015.pdf
[viii] Source: https://www.eu-patient.eu/policy/Policy/patient-empowerment/
[ix] Brothers, K. B., & Rothstein, M. A. (2015). Ethical, legal and social implications of incorporating personalised medicine into healthcare. Personalised medicine, 12(1), 43–51. https://doi.org/10.2217/pme.14.65
